# Environmental Fate, Soil Ecological Responses and Fruit Quality Impacts of Emerging Contaminants (Antibiotics) in Orchard Ecosystems: A Review

**DOI:** 10.3390/molecules31050865

**Published:** 2026-03-05

**Authors:** Yan Zeng, Wenxuan Quan, Chaochan Li

**Affiliations:** Key Laboratory for Information System of Mountainous Areas and Protection of Ecological Environment, Guizhou Normal University, Guiyang 550025, China

**Keywords:** antibiotics, antibiotic resistance genes, emerging contaminants, food safety, fruit quality, orchard soil, soil microorganisms

## Abstract

With the rapid development of intensive animal husbandry, the widespread use of livestock and poultry manure as organic fertilizers has become a major anthropogenic source of antibiotic contamination in agricultural soils. Antibiotics, classified as “emerging contaminants” owing to their persistence, biological activity, and potential ecotoxicity, undergo environmental fate processes such as adsorption–desorption, migration, transformation, and degradation upon entering orchard soils, with their behaviors regulated by multiple factors, including soil physicochemical properties, microbial communities, and climatic conditions. Antibiotics not only alter the structure and diversity of soil microbial communities, inhibit soil enzyme activities, and interfere with the cycling of carbon, nitrogen, and phosphorus nutrients but also induce the generation and dissemination of antibiotic resistance genes (ARGs) and affect the growth and reproduction of soil animals, triggering cascading effects on ecological processes. Moreover, antibiotics can be absorbed by fruit tree roots and transported to aboveground organs via the xylem or phloem. By interfering with photosynthesis, disrupting antioxidant systems, and affecting hormone balance, they inhibit the growth and development of fruit trees, thereby altering the appearance, nutritional, and flavor qualities of fruits. Furthermore, antibiotic residues and ARGs in fruits pose potential risks to food safety. This paper thoroughly analyzes the pollution levels, environmental interactions, and disposition of antibiotics in orchard soils, focusing on the mechanisms that influence their impact on soil microecology and biochemical processes. It also explores the absorption, transport, and accumulation patterns of antibiotics in fruit trees, as well as their effects on tree physiology, growth, fruit quality, and safety. Finally, the current research gaps and prospects are identified, aiming to provide a theoretical basis for ecological risk assessment, scientific prevention and control of antibiotic contamination in orchard ecosystems, and safeguarding of agricultural product safety.

## 1. Introduction

Since their initial discovery, antibiotics have played crucial roles in human health and animal husbandry [1,2]. However, this extensive application has also resulted in significant issues, particularly concerning antibiotic resistance (AMR), which has become a global public health threat [3]. It is estimated that 30% to 90% of globally prescribed antibiotics are released into the environment in their original form or as active metabolites via excreta [4,5]. This is especially important in places where animal husbandry is quite common. For example, wastewater, dung, and soils around small pig farms in northern Thailand contain 19 different types of antibiotics. The concentration of chlortetracycline in wash water was as high as 982.25 μg/L, and in manure, it was as high as 498.38 mg/kg [5]. The use of livestock manure, which is high in nutrients and organic matter on land, is a conventional and cost-effective way to fertilize soil during farming [6]. In China, orchards, especially intensive ones, have long relied on manure application to maintain soil fertility, which has unintentionally become a key pathway for antibiotics and their metabolites to enter the orchard soil ecosystem [7,8,9].

Antibiotics are persistent, biologically active, and potentially ecotoxic in the environment and are widely recognized internationally as “new pollutants” or “emerging contaminants” [10,11]. In contrast to heavy metals and persistent organic pollutants, environmental concentrations of antibiotics generally exist at trace levels (ng·kg^−1^ to μg·kg^−1^). However, their intrinsic biological activity implies that even at low concentrations (“subinhibitory concentrations”), they can induce chronic toxic effects on nontarget organisms and apply significant selective pressure, facilitating the selection and dissemination of antibiotic-resistant bacteria and resistance genes [12,13].

Orchard soil constitutes a distinct ecosystem defined by prolonged monoculture vegetation, regular agricultural interventions (such as fertilization, irrigation, and tillage), and relatively stable rhizosphere microdomains, which create a complex network of interactions among soil microbial communities, nutrient cycling, and fruit tree development [14,15,16]. The intrusion of antibiotics may disrupt this fragile balance. On the one hand, antibiotics can directly or indirectly affect the abundance, diversity, and function of soil microorganisms; alter soil enzyme activities; and interfere with the biogeochemical cycles of key elements such as carbon, nitrogen, and phosphorus, thereby affecting soil health and the nutrient supply for fruit trees [17,18]. On the other hand, antibiotics and their metabolites can be absorbed by fruit tree roots, migrating, transforming, and accumulating within the plant, potentially interfering with physiological processes (e.g., photosynthesis, antioxidant systems, and hormone balance), inhibiting growth, and ultimately affecting fruit yield, quality (e.g., sugar–acid composition, color, flavor compounds, and nutritional components), and safety [19,20].

In recent years, research on antibiotic contamination in farmland soils has increased, but most studies have focused on field crops (e.g., vegetables, rice, and wheat) [21,22]. A comparative analysis of agricultural soil antibiotic pollution-related reviews over the recent years showed that existing studies mainly focus on general farmland ecosystems, with single research dimensions (e.g., only focusing on environmental fate or ecological effects). In contrast, research on perennial woody fruit tree ecosystems is relatively fragmented and weak, lacking comprehensive integration of “environmental fate–ecological effects–plant response–food safety”. Existing studies indicate that antibiotic accumulation levels in orchard soils are often greater than those in general farmland, and long-term application of organic fertilizers containing antibiotics (e.g., fermented pig manure residue) significantly increases the abundance of antibiotic resistance genes (ARGs) in soil and on fruit surfaces, suggesting that antibiotic pollution and its risks in orchard ecosystems cannot be ignored [23]. Therefore, this paper thoroughly reviews the research progress on the environmental behavior and ecological effects of antibiotics in the “soil–plant” system of fruit trees and their impact on fruit quality, aiming to provide a reference for in-depth research in this field and a scientific basis for safe orchard production practices.

## 2. Pollution Status and Fate of Antibiotics in Orchard Soils

### 2.1. Pollution Sources and Residue Characteristics

The primary source of antibiotic pollution in orchard soils is the application of livestock manure. In intensive animal husbandry, large amounts of antibiotics are excreted via livestock waste. Posttreatment, this waste is utilized as organic fertilizer on land, serving as the primary conduit for antibiotics to infiltrate orchard soil [7,8]. Furthermore, irrigation utilizing aquaculture effluent and atmospheric deposition may provide negligible inputs; however, their overall impact remains minimal [21,22]. The types and concentrations of antibiotics in manure from various animal species fluctuate markedly, with pig manure often exhibiting the highest diversity and concentration of identified antibiotics, followed by chicken manure, whereas cattle manure presents comparatively lower levels [24,25,26].

The antibiotics identified in orchard soils primarily include tetracyclines (e.g., chlortetracycline, oxytetracycline, and doxycycline), sulfonamides (e.g., sulfamethazine, sulfamethoxazole, and sulfadiazine), quinolones (e.g., ciprofloxacin, enrofloxacin, and norfloxacin), and macrolides (e.g., azithromycin and erythromycin) (Table 1) [9,23,27]. Owing to their extensive application in livestock agriculture and gradual degradation, tetracyclines are the most prevalent antibiotic contaminants in orchard soils [18,19]. Residue concentrations change across various orchard types. Antibiotic residues in the soils of intensive orchards, such as citrus and apple orchards, are generally elevated compared with those in conventional orchards, mostly due to variables such as increased manure application rates and extended planting cycles in intensive systems [9,23,27]. For example, the cumulative residue of tetracycline antibiotics in citrus orchard soils in southern China can reach levels of 10–50 μg/kg, with some high-yield orchards exceeding 100 μg/kg, while those in farmland soil are mostly <20 μg/kg [9]; the prevalence of sulfonamide antibiotics in apple orchard soils is 100%, with sulfamethazine residues reaching as high as 32.8 μg/kg, with farmland soil mostly <10 μg/kg [23]. The higher antibiotic residue levels in orchard soils are mainly attributed to long-term application of organic fertilizer, perennial continuous cropping, and high organic matter adsorption and retention capacity of orchard soils [28,29,30,31,32,33].

**Figure 1:**
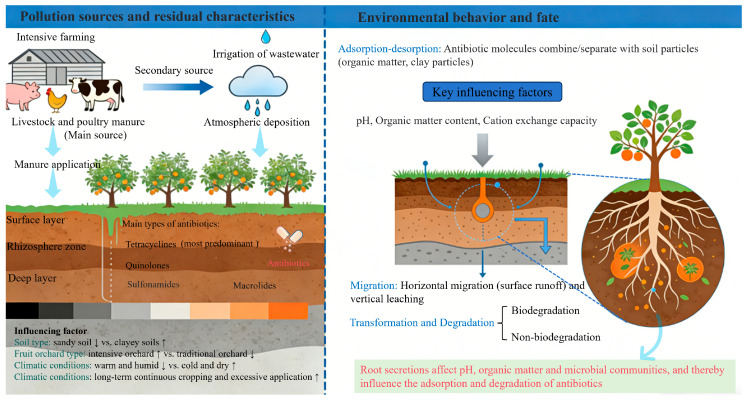
Pollution sources and residual characteristics, and environmental behavior and fate of antibiotics in orchard soils. Antibiotics primarily enter the soil through the application of livestock manure (with varying contributions from pig, chicken, and cattle), with minor inputs from wastewater irrigation and atmospheric deposition. The main antibiotic classes detected include tetracyclines, sulfonamides, quinolones, and macrolides. Their environmental behavior, encompassing adsorption–desorption, migration (horizontal transport and vertical leaching), and degradation (biotic and abiotic pathways), is regulated by soil properties (e.g., organic matter, clay content, pH, and CEC), climate conditions, and microbial activity. The rhizosphere microdomain, influenced by root exudates, serves as a hotspot for these processes.

The properties of antibiotic residues in orchard soils are regulated by soil type, climatic conditions, and management strategies (Figure 1). Antibiotics have stronger leaching and migration capabilities in sandy soils, leading to relatively lower residues [34,35]. Clayey soils and soils with high organic matter contents have strong adsorption capacities for antibiotics, resulting in higher residues [36,37]. Warm and humid climates favor the biodegradation of antibiotics, whereas cold and dry regions have longer antibiotic persistence [38,39]. Soils under long-term continuous cropping and excessive manure application show significant accumulation effects, with residue levels significantly increasing with increasing planting duration [23,40]. However, current residue detection methods have limitations, such as low sensitivity for trace antibiotic detection (difficult to detect ng/kg level residues) and lack of simultaneous detection of metabolites, which may lead to underestimation of actual pollution levels [35].

### 2.2. Environmental Behavior and Fate

After entering orchard soil, antibiotics undergo a series of environmental behaviors, including adsorption–desorption, migration, transformation, and degradation, processes regulated by soil physicochemical properties, microbial communities, climate conditions, and other factors (Figure 1) [4,7,20]. The environmental fate of antibiotics is determined by the coupling of biodegradation and abiotic degradation: biodegradation is the core process of antibiotic elimination, while abiotic degradation acts as a supplementary pathway, and the two processes interact and jointly affect the persistence and migration of antibiotics in soil [11,20].

#### 2.2.1. Adsorption–Desorption

Adsorption–desorption is a prerequisite for antibiotic migration and transformation in soil. The soil organic matter content, clay content, pH, and cation exchange capacity (CEC) are key factors affecting the adsorption process. Soil organic matter (SOM) primarily sequesters antibiotics via hydrophobic partitioning, particularly for non-polar molecules, but can also engage in hydrogen bonding and π-π interactions [36,41]. Clay minerals, on the other hand, leverage their high specific surface area and permanent negative charge to adsorb antibiotics strongly through cation exchange and electrostatic attraction, especially for species that exist as cations (e.g., tetracyclines under acidic conditions) [41,42]. Soil pH indirectly influences adsorption efficiency by affecting the dissociation state of antibiotics and the surface charge of soil colloids [43,44], through three core mechanisms: (i) Regulation of the dissociation form of antibiotic molecules: under low pH conditions, antibiotics such as tetracyclines and sulfonamides predominantly occur in neutral or cationic forms, whereas at elevated pH values, they predominantly become anionic [45,46]; (ii) Influence on the surface charge of soil colloids: soil colloids (organic matter and inorganic minerals) are generally negatively charged under neutral to alkaline conditions, and positively charged under strongly acidic conditions, leading to changes in electrostatic interactions with antibiotic molecules [47,48]; (iii) Modulation of binding modes: pH affects the strength of hydrophobic interactions and hydrogen bonding between antibiotics and soil organic matter. For example, under acidic conditions, the protonation of amino groups in tetracyclines enhances hydrogen bonding with soil organic matter, promoting adsorption; under alkaline conditions, the deprotonation of hydroxyl groups weakens this interaction, reducing adsorption efficiency [48,49].

#### 2.2.2. Migration and Degradation

Mobility is an important indicator of the environmental risk of antibiotics. Antibiotic migration in soil mainly includes horizontal transport and vertical leaching [50]. The mobility of different antibiotics varies significantly, which is closely related to their adsorption capacity [51,52,53]. The adsorption capacity of different antibiotics can be quantified by the soil-water distribution coefficient (Kd), with values spanning several orders of magnitude, which directly reflects their potential mobility risk [41,43]. For instance, under typical acidic–neutral, moderate-OC agricultural soils, both tetracyclines and fluoroquinolones usually have Kd in the 10^2^–10^3^ (up to 10^4^) L/kg range [28,29,30]. Such Kd values correspond to strong adsorption and weak mobility in soil, which is expected to result in minimal leaching compared with weakly sorbed antibiotics (e.g., sulfonamides with Kd often <5 L/kg) [29]. While the reported Kd value of macrolide antibiotics in agricultural soils spans roughly 2–130 L/kg, with tylosin occasionally reaching values above 100 L/kg in organic-rich soils, clarithromycin typically shows Kd values of 2.5–10.5 L/kg [29,32,33]. These values indicate moderate sorption and appreciable mobility compared with strongly sorbing tetracyclines and fluoroquinolones but lower mobility than sulfonamides.

Antibiotics undergo transformation and degradation in orchard soil through biotic and abiotic mechanisms. Biodegradation, which is chiefly facilitated by soil microorganisms such as bacteria and fungi by processes such as oxidation–reduction, hydrolysis, and dehalogenation, is the principal mechanism for antibiotic elimination, converting them into nontoxic or less toxic byproducts [11,20]. The rates of biodegradation differ markedly among antibiotics. Sulfonamides exhibit rapid degradation, with half-lives often ranging from days to weeks; in contrast, tetracyclines and quinolones degrade more slowly, with half-lives extending to months or even years [4,18]. Abiotic degradation encompasses photodegradation, hydrolysis, and redox processes. Photodegradation facilitates the elimination of antibiotics in surface soil, whereas hydrolysis and redox processes are affected by soil pH and redox potential, among other factors. [7,35].

#### 2.2.3. Special Effects of Orchard Rhizosphere Microdomain

The rhizosphere microdomains of orchards are active areas for antibiotic migration and transformation, and are the hotspots of antibiotic adsorption–degradation and ARG dissemination in orchard soil systems [20,54]. Root exudates (e.g., organic acids, amino acids, sugars) of fruit trees can modify the rhizosphere soil pH, organic matter composition, and microbial community structure, thereby influencing antibiotic adsorption and desorption mechanisms [20,54]. Certain root exudates may serve as carbon sources for soil microorganisms, promoting the growth and reproduction of antibiotic-degrading bacteria and thus facilitating antibiotic breakdown [20,54]. Fruit tree root exudates promote the physical contact between rhizosphere microorganisms and activate the quorum-sensing system of microorganisms, which significantly accelerates the horizontal transfer of antibiotic resistance genes (ARGs) in the rhizosphere [55,56]. The unique ecological conditions of orchard rhizosphere (stable microenvironment, high microbial abundance, and continuous secretion of root exudates) make its ARG dissemination efficiency significantly higher than that of non-rhizosphere soil [23,57].

## 3. Ecological Impacts of Antibiotics on Orchard Soil Ecosystems

### 3.1. Effects on Soil Microbial Community Composition and Diversity

Soil microbial communities are the foundation of soil ecosystems, engaging in essential processes such as organic matter breakdown and nutrient cycling. Structural stability and diversity are essential for soil health [58,59]. Antibiotics applied to orchard soils impose selective pressure on soil microbial communities, resulting in notable alterations in microbial abundance, diversity, and community structure [60,61]. Environmentally relevant concentrations/sub-inhibitory concentrations (low levels, tetracyclines: 1–10 μg/kg; sulfonamides: 0.5–5 μg/kg) of antibiotics in soils do not induce significant microbial mortality; nonetheless, they can suppress the proliferation of susceptible bacteria, facilitate the enhancement of antibiotic resistance genes (ARGs) and the emergence of drug resistance, and modify the microbial community structure and function [62,63]. Concentrations exceeding soil ecotoxicological thresholds (high levels, tetracyclines: >100 μg/kg; sulfonamides: >50 μg/kg) of antibiotics markedly diminish total soil microbial biomass, alter community structure, reduce diversity, impede microbial metabolic activity and ecological functions, and adversely affect soil enzyme activities, effects that are more pronounced in soils with low organic matter content [18,64]. Elevated levels of antibiotics (e.g., tetracyclines: >100 μg/kg; sulfonamides: >50 μg/kg) result in a significant reduction in bacterial populations, promote the development of drug-resistant strains, and increase ecological risk, and their residues and migration in soils can perpetually influence the restoration of microbial communities and fluctuations in resistance genes [61,62].

The impact of antibiotics on soil microbial diversity depends on the antibiotic concentration, particularly the species [18,23]. Low concentrations may promote tolerant microbial groups, sustaining or marginally enhancing diversity, whereas high concentrations markedly suppress overall microbial abundance, especially impacting Gram-positive bacteria and fungi, and induce alterations in community structure and function [60,65,66,67]. This concentration-dependent process is intricately associated with the ecotoxicological threshold of antibiotics. Once ambient concentrations surpass the critical value, microbial stress responses or mortality are induced [66]. Different orchard types show significant differences in the response of soil microbial communities to antibiotics: citrus orchard soils in southern China with high organic matter content have stronger buffering capacity, and the inhibitory effect of antibiotics on microbial communities is weaker than that of apple orchard soils in northern China [9,23]. Various antibiotics impose unique selective pressures on microbial populations. For instance, quinolones (e.g., OFL, CIP) may increase the growth of particular groups such as Proteobacteria, whereas sulfonamides (e.g., SDZ) augment the reservoir of resistance genes via mobile genetic elements linked to their targets and the intrinsic resistance mechanisms of microorganisms [66,68,69]. At the community level, antibiotics promote resistant taxa (e.g., Proteobacteria and Firmicutes), suppress sensitive taxa (e.g., Acidobacteria and Actinobacteria), and decrease the prevalence of functional genes linked to organic matter breakdown and nitrogen transformation [15,17,18,23].

Existing studies on the effects of antibiotics on orchard soil microbial communities are mostly based on short-term indoor simulation experiments, and there is a lack of long-term in situ monitoring research; meanwhile, most studies focus on the effects of a single antibiotic, and the research on the combined effects of multiple antibiotics is scarce, which cannot reflect the actual pollution situation of orchard soils.

### 3.2. Disruption of Soil Enzyme Function and Nutrient Cycling

Soil enzymes resulting from microbial metabolism facilitate the transformation of soil nutrients, including carbon, nitrogen, and phosphorus. Their activities may indicate the efficacy of organic matter decomposition and nutrient cycling in soils [70,71]. The impact of antibiotics on soil enzyme activity is intricate and varied and is influenced by parameters such as antibiotic type, concentration, soil type, and enzyme type [7,18]. Antibiotics typically decrease soil enzyme activities by suppressing microbial activity or directly interacting with enzyme proteins; however, low dosages may marginally increase certain enzyme activities by prompting bacteria to generate resistance enzymes [18,72,73]. Moreover, antibiotics interact with environmental variables, including soil texture and temperature, to affect the extent of enzyme inhibition; greater inhibition is observed in fine-textured soils and neutral pH conditions [60]. The amalgamation of antibiotics and heavy metal contamination generates synergistic inhibitory effects, intensifying the detrimental consequences on soil enzyme activities [74].

Antibiotics indirectly affect the cycling of key nutrients (carbon, nitrogen, and phosphorus) in orchard soils by regulating enzyme activities, interfering with functional gene expression, and altering the microbial community structure [18,75]. In nitrogen cycling, antibiotics impede the expression of functional genes such as *ureC*, *nirK*, and *norB*, obstructing urea decomposition and denitrification processes, which diminishes nitrogen availability and plant absorption efficiency [76]; additionally, tetracyclines such as oxytetracycline inhibit urease activity, thereby decreasing the mineralization rate of soil organic nitrogen and reducing the available nitrogen content, consequently hindering fruit tree growth [77,78]. Antibiotic stress during phosphorus cycling markedly diminishes soil phosphatase activity (both acid and alkaline phosphatases), hindering the transformation of organic phosphorus into accessible phosphorus and decreasing the bioavailability of soil phosphorus [78,79]. Furthermore, antibiotic disruption of soil microbial populations impacts carbon cycling processes, diminishing the breakdown rate of soil organic matter and resulting in organic matter buildup or a decrease in quality [80,81]. Over time, the disruption caused by antibiotics to soil enzyme activity and nitrogen cycling leads to diminished soil fertility, consequently impacting nutrient availability and the growth of fruit trees.

The response of soil nutrient cycling to antibiotics in different orchard types is different: the high organic matter content and abundant microbial communities in citrus orchard soils make the nutrient cycling process more stable, and the interference effect of antibiotics on carbon, nitrogen and phosphorus cycling is weaker than that in apple orchard soils [9,75].

### 3.3. Induction and Dissemination of ARGs

**Figure 2:**
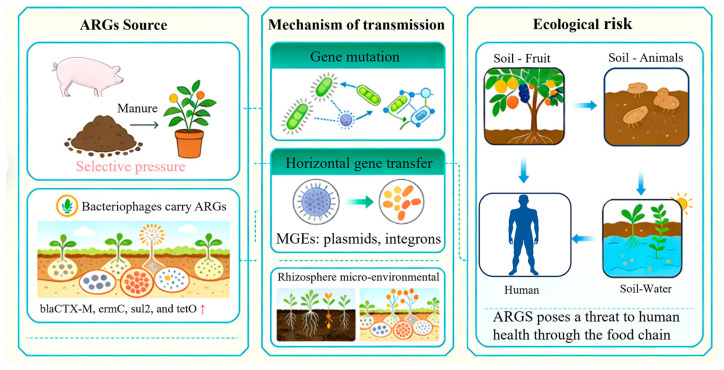
Ecological impacts of antibiotics on the soil microbiome and the proliferation of antibiotic resistance genes (ARGs). Antibiotics exert selective pressure on soil microbial communities, inhibiting sensitive taxa while enriching resistant ones, thereby reducing diversity and altering community structure. Critically, antibiotics induce the generation and horizontal gene transfer (HGT) of antibiotic resistance genes (ARGs) via mobile genetic elements (MGEs) like plasmids and integrons. The rhizosphere, with its active microbial community and root exudates, acts as a key hotspot for ARG dissemination between soil, plants, animals, and water, posing a risk to food safety.

ARGs are functional genes that confer resistance to certain antibiotics in bacteria, and their spread in the environment has emerged as a significant global concern (Figure 2) [82,83]. Long-term selective pressure from antibiotics in orchard soils, particularly through the application of antibiotic-containing livestock manure, significantly increases the abundance of ARGs, which is positively correlated with soil antibiotic concentrations [23,84]. Research indicates that the prolonged utilization of anaerobic fermentation residues from pig manure in apple orchards markedly elevates the relative abundance of ARGs, such as *blaCTX-M*, *ermC*, *sul2*, and *tetO*, in soils. These ARGs propagate in soils and on fruit surfaces via mobile genetic elements, thereby increasing the degree of ecological safety risk [23]. Furthermore, ARGs in orchard soils are present not only in bacteria but also in phages, with a notable increase in phage-associated ARGs, particularly in soils amended with organic fertilizers, suggesting that various carriers collaboratively enhance ARG dissemination [85].

#### 3.3.1. Sub-MIC/MSC Selection Pressures

Sub-inhibitory concentration (sub-MIC) of antibiotics is an important driving factor for the induction and enrichment of ARGs in orchard soils. For instance, sub-MIC concentrations of sulfonamides (0.1–1 μg/kg) can increase the abundance of the *sul2* gene in apple orchard soil by 1.5–3.0 times, and sub-MIC concentrations of tetracyclines (1–5 μg/kg) can increase the abundance of the *tetO* gene by about 2 times [23,56,66]. The sub-MIC of antibiotics can induce the expression of resistance-related genes in soil microorganisms, promote gene mutation and horizontal transfer, and thus enhance the resistance of microbial communities to antibiotics [66,86]. The continuous input of antibiotics in orchard soils (via organic fertilizer application) maintains a long-term sub-MIC selective pressure, which is the main reason for the high abundance of ARGs in orchard soils compared with general farmland soils [23,84].

#### 3.3.2. Horizontal Gene Transfer (HGT) Mechanisms

Antibiotics induce ARGs in soils through two primary mechanisms: first, they apply selective pressure on bacteria, leading to gene mutations that generate new resistance genes; second, HGT promotes the spread of existing ARGs among bacterial populations [86,87]. The three main HGT mechanisms (conjugation, transduction, and transformation) play important roles in the dissemination of ARGs in orchard soils. Research indicates that mobile genetic elements (MGEs), including plasmids and integrons, are pivotal in the horizontal transfer of ARGs within the rhizosphere; the structure of the rhizosphere microbial community and its metabolites (e.g., indole compounds) govern the dissemination of ARGs [55,88]. Additionally, microbial community turnover in the plant rhizosphere and antibiotic pressure collaboratively facilitate the enrichment and dissemination of particular ARGs, especially in soils containing antibiotics [56]. Experimental simulations have demonstrated that strains carrying ARGs can successfully colonize the rhizosphere and transfer ARGs to rhizosphere microbial communities through conjugation [57,89]. The active metabolism of rhizosphere microorganisms and root exudates not only promotes physical contact between microorganisms but also may inhibit the transfer of certain ARGs, demonstrating a complex regulatory mechanism [55,56]. The rhizosphere of fruit trees is home to many microbes and is very active. Root exudates help microbes touch and talk to each other, which makes the transfer of ARGs much more efficient [56,57]. Owing to their unique ecological conditions and microbial activity, the rhizosphere microenvironment of orchard soils is a hot spot for horizontal ARG transfer. For instance, the conjugative transfer frequency of the *tetO* gene mediated by plasmids in the rhizosphere of apple orchards is 2–4 times higher than that in non-rhizosphere areas [23].

#### 3.3.3. ARG Dissemination in the Orchard Ecosystem

The dissemination of ARGs in orchard soils not only alters the resistance characteristics of soil microbial communities but also may pose threats to human health through the food chain (Figure 2). Fruit tree root systems can adsorb soil-borne antibiotic-resistant bacteria (ARB), but these bacteria rarely enter plant tissues. ARGs are primarily present on the fruit surface (detection rate 30–60%), with internal detection rates < 5% [84,90,91]. Soil ARGs can also disseminate through intermediate hosts such as soil animals (e.g., earthworms) and insects, ultimately entering the human body [89,90,92]. Additionally, ARGs may enter aquatic environments through farmland irrigation and surface runoff, expanding the degree of pollution [7,11]. It is necessary to conduct more research on the exact ways that ARGs build up in fruits, but current models and experiments show that root absorption and soil animal-mediated dissemination are important ways for ARGs to enter the food chain [89,90]. Therefore, it is very important to control how ARGs spread in soils and how fruit trees absorb them to lower the risks to human health that come from the food chain.

### 3.4. Cascading Effects on Soil Fauna and Ecological Processes

Soil animals are important components of soil ecosystems and participate in processes such as soil structure formation, organic matter decomposition, and nutrient cycling. The stability of their community structure and function is crucial for soil ecological balance [93,94]. Earthworms, among the most abundant and functionally important soil animals in orchards, are relatively sensitive to antibiotics. Research indicates that prevalent antibiotics, including tetracycline, ciprofloxacin, and sulfamethoxazole, do not induce acute toxicity in earthworms at environmentally relevant concentrations; however, prolonged exposure hampers growth, disrupts physiological and biochemical functions, and may even compromise reproductive capacity [95,96]. Antibiotics also change the microbial community in the intestines of earthworms, making bacteria that carry resistance genes grow faster and making potential pathogens more common [95,97]. Antibiotics also have strong effects on small soil animals, such as nematodes and collembolans. For example, high levels of antibiotics decrease the diversity of nematode communities, increase the number of plant-parasitic nematodes, and decrease the number of predatory and saprophytic nematodes [98,99].

Changes in the communities of soil animals have a chain effect on the ecological processes in the soil. A reduction in the number of large soil animals, such as earthworms, decreases soil aeration and water permeability, thereby affecting soil structure formation and plant root growth [100,101,102]. Alterations in nematode community structure interfere with interactions between soil microbial communities and plants, indirectly affecting nutrient cycling processes [103,104,105]. Furthermore, as intermediate links in the food chain, the accumulation and transformation of antibiotics in soil animals affect the biogeochemical cycling of pollutants and may affect higher trophic levels through biomagnification [81,106]. Presently, research regarding the cascading effects of antibiotics on orchard soil fauna and ecological processes is somewhat limited and requires additional investigation, especially the research on the combined effects of multiple antibiotics on soil animal communities and the long-term cascading effects on orchard ecosystem functions.

## 4. Impact of Antibiotics on Fruit Tree Growth and Fruit Quality

### 4.1. Plant Absorption, Movement, Storage and Transformation Products

The absorption, translocation, and accumulation of antibiotics by fruit trees are prerequisites for their impacts on fruit quality and food safety, a process influenced by factors such as antibiotic type, soil properties, and fruit tree variety [19,35,107,108]. Antibiotics enter roots primarily through passive diffusion (driven by concentration gradient, main pathway for lipophilic antibiotics such as macrolides) or active transport (driven by membrane transporters, main pathway for hydrophilic antibiotics such as tetracyclines) across the epidermal cells of fruit tree roots and are subsequently translocated to aboveground organs such as stems, leaves, and fruits through the xylem or phloem [107,109,110]. Different antibiotics have very different abilities to be absorbed and moved around. In general, fruit trees can absorb and move around antibiotics that are very hydrophobic and have small molecular weights (such as sulfonamides and macrolides) more easily. On the other hand, antibiotics that are very hydrophilic and have large molecular weights (such as tetracyclines and quinolones) mostly accumulate in the roots and do not move around as easily to aboveground organs [35,108,111].

Fruit trees show different levels of antibiotic accumulation in different parts of the tree. Specifically, roots tend to have the greatest accumulation, followed by stems and leaves, while fruits have the least accumulation [35,109]. Different fruit tree varieties have significant differences in the absorption and accumulation of antibiotics. For example, tetracyclines in litchi trees primarily accumulate in the roots, with negligible translocation to the fruits; no antibiotic residues were found in the fruits following prolonged application of antibiotic-containing organic fertilizers [19]. In citrus trees, oxytetracycline and streptomycin applied via injection or root application were detected in roots, stems, and leaves, but fruit concentrations were low [109,112]. Some antibiotics are very stable in plants [109,112]. Even though the levels of antibiotics in fruit are low (mostly <0.1 μg/kg), there are still possible food safety risks, especially in orchards where antibiotic-containing organic fertilizers or pesticides have been used for a long time. This is because antibiotics and their resistance genes can spread through soil–plant chains [23,84]. Detection technologies such as liquid chromatography-high-resolution mass spectrometry (LC-HRMS) have been used for the sensitive detection of multiple antibiotic residues in fruits, ensuring concentrations below safety standards [35,113]. Therefore, even if fruits do not contain many antibiotics, their safety risks cannot be ignored. This means that orchard management and antibiotic use need to be watched more closely.

The main changes associated with antibiotics in fruit trees are oxidation, reduction, hydrolysis, and conjugation reactions. These reactions make metabolites that are less or more toxic [108,114]. The main transformation products of tetracyclines in fruit trees are oxidation products (dehydrotetracycline) and hydrolysis products (anhydrotetracycline), and the main transformation products of sulfonamides are acetylation products and oxidation products; some of these transformation products exhibit toxicity comparable to their parent compounds [108,113]. Research indicates that antibiotics such as tetracyclines and penicillins undergo structural alterations via enzymatic reactions in plants, resulting in metabolites that may exhibit diminished toxicity in certain instances while potentially producing novel harmful compounds [108,113]. For example, when penicillin and oxytetracycline are injected into citrus trees, they break down partially in vivo, resulting in low levels of metabolites that do not have much of an effect on fruit safety [112]. However, more research is needed on specific metabolic pathways and the ecotoxicological effects of metabolites [112,115]. At present, research regarding the transformation mechanisms of antibiotics in fruit trees is insufficient; subsequent studies should concentrate on metabolite identification and toxicological assessment to comprehensively elucidate the potential effects of antibiotics on fruit trees and fruit safety.

### 4.2. Toxic Effects on Fruit Tree Physiological Metabolism and Growth Development

**Figure 3:**
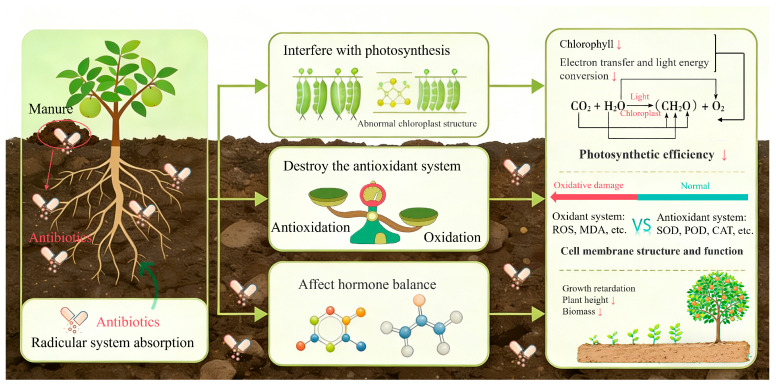
Pathways and effects of antibiotics within fruit trees. Antibiotics are absorbed by roots. Inside the plant, antibiotics interfere with key physiological processes: they inhibit photosynthesis by damaging chloroplasts and photosystem II (PSII), induce oxidative stress (leading to ROS accumulation and membrane damage indicated by MDA), and disrupt hormone (e.g., IAA, GA) balance. These toxic effects ultimately impact fruit tree growth and development.

Once absorbed by fruit trees, antibiotics exert toxic effects on physiological metabolism and growth, primarily by disrupting photosynthesis, antioxidant systems, and hormone balance (Figure 3) [116,117,118]. Photosynthesis is the foundation for fruit tree growth and development; antibiotics reduce photosynthetic efficiency by inhibiting photosynthetic pigment synthesis or damaging the photosynthetic organ structure. Studies have shown that tetracyclines and sulfonamides decrease chlorophyll levels, obstruct chlorophyll biosynthesis pathways, induce leaf chlorosis and decrease the rate of photosynthesis [119]. Additionally, antibiotics such as quinolones target photosystem II (PS II) core proteins, interfering with electron transfer and light energy conversion and further impairing the light reaction stage of photosynthesis [120]. Antibiotic treatment also causes chloroplasts to swell and damage their thylakoid membranes, which affects how well photosynthetic organs work [121]. These changes make photosynthesis less efficient, resulting in a lower net photosynthetic rate and photosynthetic yield. This affects plant growth and yield.

Antibiotic stress triggers oxidative stress responses in fruit trees, resulting in the excessive accumulation of reactive oxygen species (ROS) and subsequent damage to the cellular membrane structure and function [121,122]. To resist oxidative damage, fruit trees activate antioxidant systems, increasing the activity of antioxidant enzymes such as superoxide dismutase (SOD), peroxidase (POD), and catalase (CAT), as well as the content of nonenzymatic antioxidants such as glutathione and ascorbic acid [122,123,124]. For instance, low-concentration tetracycline (1–10 μg/kg) causes a 10–15% increase in SOD enzyme activity of litchi leaves [19,123,124]. However, high concentrations of antibiotics exceed the regulatory capacity of the antioxidant system, leading to increased malondialdehyde (MDA) content, increased cell membrane permeability, and the appearance of symptoms of oxidative damage [122,123]. For example, high-concentration tetracycline (>100 μg/kg) leads to a more than 50% increase in MDA content and a 30–40% inhibition rate of litchi root length [19,123,124], increased cell membrane permeability, and the appearance of symptoms of oxidative damage [122,123]. Furthermore, antibiotics further exacerbate ROS production by interfering with photosynthesis and the mitochondrial electron transport chain, affecting plant energy metabolism and growth [121,125].

Antibiotics also affect the hormone balance of fruit trees, interfering with growth and development processes [117]. Gibberellins (GA) and auxins (IAA) are key hormones that promote fruit tree growth and fruit development; antibiotics inhibit their synthesis or promote their decomposition, leading to stunted growth, reduced plant height, and decreased biomass [126,127]. Antibiotics also affect the flowering and fruiting of fruit trees, causing delayed flowering, a reduced fruit set rate, and poor fruit development [116,128]. Additionally, antibiotics significantly inhibit the growth of fruit tree roots, resulting in a shortened root length, a reduced root surface area, and a decreased lateral root number, affecting root water and nutrient absorption [124,129]. Different fruit tree varieties have significant differences in the physiological response to antibiotics: litchi trees are more sensitive to tetracycline stress than citrus trees, and the oxidative damage and growth inhibition caused by the same concentration of tetracycline in litchi trees are more severe [19,112].

### 4.3. Impact on Fruit Quality and Food Safety

The impacts of antibiotics on fruit quality are reflected in three main aspects: appearance quality, nutritional quality, and flavor quality. Antibiotic treatment can increase fruit size, thicken fruit peel, or alter color but may also cause irregular fruit shape and uneven color, affecting commercial value; for example, antibiotic treatment of citrus fruits increases peel thickness and decreases juice yield [116,130]. In terms of nutritional quality, antibiotic application generally increases fruit vitamin C, total sugar, and phenolic compound contents, increasing antioxidant capacity, but high concentrations may cause nutritional imbalances and reduce vitamin C contents [116,130,131]. In terms of flavor quality, appropriate antibiotic treatment helps increase fruit sugar content and soluble solid content, improving taste, but excessive antibiotic use inhibits normal fruit metabolism and reduces the synthesis of flavor substances [116,131].

In addition to antibiotic residues, the accumulation of antibiotics in fruits is accompanied by ARGs and drug-resistant bacteria, posing risks to food safety and public health [91,131]. Studies have detected various ARGs on the surface and inside fruits, such as grapes; these genes may disseminate through intestinal microorganisms, increasing the risk of drug-resistant flora in the human body [11,91]. Based on existing fruit residue data (mostly <0.1 μg/kg), the estimated daily intake (EDI) values of antibiotics from fruits were calculated, showing results far below the World Health Organization (WHO)’s specified acceptable daily intake (ADI) values, indicating low acute health risks [91,132]. However, long-term intake risks and ARG transmission risks require attention [91,132]. Although short-term low-dose exposure has limited physiological impacts on animals, long-term or high-dose intake may increase the abundance of resistance genes in intestinal microorganisms, affect digestive and absorption functions, and reduce the clinical efficacy of antibiotics [91,133]. Furthermore, antibiotic residues may trigger allergic reactions, microbial dysbiosis, and immune function changes, increasing health risks [132,134]. Currently, there are no unified maximum residue limit (MRL) standards for antibiotics in fruit trees domestically and internationally, a significant research gap that limits the comprehensive risk assessment of antibiotic residues in fruits. Research on long-term health impacts of low-dose antibiotic intake from fruits is also insufficient, requiring enhanced supervision and scientific research.

## 5. Conclusions, Shortcomings, and Future Prospects

### 5.1. Conclusions

This paper thoroughly reviewed the pollution status, environmental behavior, and fate of antibiotics in orchard soils and their effects on the soil environment, fruit tree growth, and fruit quality. Research indicates that livestock manure application is the primary source of antibiotic pollution in orchard soils. Tetracyclines, sulfonamides, quinolones, and macrolides are the main antibiotics detected, with residue levels in intensive orchards being significantly higher than those in traditional orchards. Antibiotics undergo fate processes such as adsorption–desorption, migration, transformation, and degradation in orchard soil, and their behavior is regulated by soil physicochemical properties and microbial communities.

**Figure 4:**
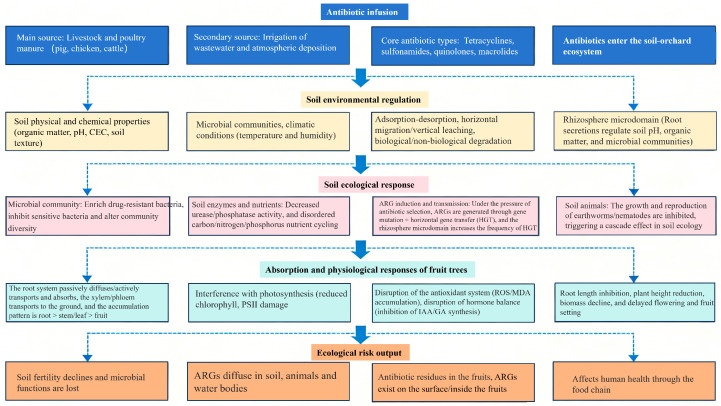
Conceptual framework of antibiotic–soil–plant–microbial community interactions and their multi-dimensional impacts in orchard ecosystem.

Antibiotics have significant ecological effects on the orchard soil environment, primarily manifested as: (1) altering the soil microbial community structure and diversity, enriching resistant microbial taxa while inhibiting sensitive microbial taxa; (2) reducing soil enzyme activity and interfering with carbon, nitrogen, phosphorus, and other nutrient cycling processes; (3) inducing ARG production and dissemination, increasing environmental risks; and (4) affecting the growth and reproduction of soil fauna, triggering cascading ecological effects. Moreover, antibiotics can be absorbed, translocated, and accumulated within fruit trees. By interfering with photosynthesis, disrupting antioxidant systems, and affecting hormone balance, they inhibit fruit tree growth and development and reduce fruit appearance and nutritional and flavor quality. Furthermore, antibiotic residues in fruits pose potential threats to food safety (Figure 4).

### 5.2. Research Shortcomings

Although progress has been made in research on antibiotic pollution in orchard soils, several shortcomings remain: 

(1) Most existing studies focus on the pollution characteristics and effects of single or a few antibiotics; research on the combined effects of composite antibiotic pollution is insufficient, and attention to newer antibiotics (e.g., cephalosporins, carbapenems) is limited. Most studies are based on short-term indoor simulation experiments, lacking long-term in situ monitoring research on antibiotic pollution in orchard soils.

(2) The transformation mechanisms of antibiotics within fruit trees, the types of metabolites, and their toxic effects are still unclear, lacking systematic research; the dissemination pathways of ARGs in the orchard soil–plant–animal system are not fully clarified, there are no unified MRL standards for antibiotics in fruits, and the long-term health risk assessment system is not perfect.

(3) The research on the cascading effects of antibiotics on orchard soil fauna and ecological processes is scarce; the research on the combined effects of antibiotics and other pollutants (heavy metals, pesticides) on orchard soil microecology is insufficient; and the molecular mechanism of ARG dissemination in the orchard rhizosphere microdomain needs to be further explored.

(4) Research on remediation technologies for antibiotic pollution in orchard soils is relatively weak, lacking economically efficient and environmentally friendly prevention and control measures; the research on the tolerance differences and resistance mechanisms of different fruit tree varieties to antibiotics is insufficient, limiting the screening and utilization of resistant germplasm resources.

(5) The research on the coupling mechanism of antibiotic biodegradation and abiotic degradation in orchard soils is not in-depth; the migration law of antibiotics in different orchard soil types (sandy soil, clayey soil) and different soil layers needs to be further clarified; and the research on the regulatory mechanism of fruit tree root exudates on antibiotic environmental behavior is relatively weak.

### 5.3. Future Prospects

Future research should focus on the following five dimensions, and propose targeted research directions for each key research gap:

(1) Pollution characteristics: Strengthen studies on the environmental behavior and ecological effects of composite antibiotic pollution and newer antibiotics (cephalosporins, carbapenems), clarify their combined toxicity mechanisms, and improve the pollution assessment system; carry out cross-regional and long-term in situ monitoring surveys across multiple orchard types (citrus, apple, and litchi), and clarify the spatial and temporal variation characteristics of antibiotic pollution in orchard soils.

(2) Environmental behavior: Study the coupling mechanism of antibiotic biodegradation and abiotic degradation in orchard soils in depth, and explore the effects of soil physicochemical properties and microbial communities on the coupling process; clarify the migration law of antibiotics in different orchard soil types and soil layers, and establish a migration prediction model; and systematically study the regulatory mechanism of fruit tree root exudates on antibiotic adsorption, degradation and migration in the rhizosphere microdomain.

(3) Ecological effects: Carry out in-depth research on the cascading effects of antibiotics on orchard soil fauna and ecological processes, and clarify the impact of antibiotic pollution on the structure and function of orchard soil food webs; strengthen the research on the combined effects of antibiotics and other pollutants on orchard soil microecology, and clarify the synergistic/antagonistic effect mechanism; and explore the molecular mechanism of ARG dissemination in the orchard rhizosphere microdomain driven by root exudates and quorum sensing systems, and establish an ARG dissemination risk assessment model.

(4) Food safety: Utilize multi-omics technologies to deeply analyze the transformation pathways, metabolite toxicity, and molecular response mechanisms of antibiotics within fruit trees; systematically study the dissemination patterns of ARGs in the orchard soil–plant–animal system, and clarify the key transmission nodes and driving factors; and accelerate the formulation of MRL standards for antibiotics in fruits, and establish a comprehensive food safety risk assessment system including acute and long-term health risks.

(5) Control and prevention technologies: Develop economically efficient and environmentally friendly combined remediation technologies based on microbial remediation, phytoremediation, and immobilization remediation, and explore the application effect of these technologies in orchard soils; explore agricultural management practices that reduce antibiotic input and pollution risk (e.g., optimizing organic fertilizer application mode, planting cover crops); conduct screening for antibiotic tolerance among different fruit tree varieties, mine resistance-related genes, and provide germplasm resources and theoretical support for breeding resistant varieties; and establish a comprehensive prevention and control system for antibiotic pollution in orchard ecosystems integrating “source control–process interception–end remediation”. In addition, strengthen the application of emerging research methods such as Hydrus-1D (HYDRUS) Model and Well Head Protection Area (WHPA) Model for predicting antibiotic migration, multi-omics for analyzing molecular mechanisms, and integrated frameworks for holistic assessment across the ‘Environmental Fate–Ecological Effects–Food Safety’ chain, and clarify the application limitations and optimization directions of these methods in orchard antibiotic pollution research.

## Figures and Tables

**Table 1:** Key physicochemical properties of typical antibiotics in orchard soils.

Antibiotic Class	Typical Representative	Typical Soil Kd Trend	Dominant Interactions in Soil	Mobility/Leaching Risk	References
Tetracyclines	chlortetracycline, oxytetracycline, and doxycycline	Highest (10^2^–10^3^ L/kg; (up to 10^4^))	Cation exchange, metal chelation, surface complexation	Very low; accumulate in topsoil	[28,29,30]
Quinolones	ciprofloxacin, enrofloxacin, and norfloxacin	High (10^2^–10^3^ L/kg)	Cation exchange, electrostatic sorption	Low; strong sediment/soil binding	[28,31]
Macrolides	azithromycin and erythromycin	Moderate (10^1^–10^2^ L/kg)	Hydrophobic + ionic interactions	Moderate	[28,29,32]
Macrolides	sulfamethazine, sulfamethoxazole, and sulfadiazine	Lowest (≈1–10 L/kg)	Weak hydrophobic/H-bonding to OM	Highest; prone to groundwater/porewater contamination	[28,29,32,33]

## Data Availability

No new data were created or analyzed in this study.

## Abbreviations

The following abbreviations are used in this manuscript:

ADI	Acceptable daily intake
AMR	Antibiotic resistance
ARGs	Antibiotic resistance genes
CAT	Catalase
CEC	Cation exchange capacity
EDI	Estimated daily intake
GA	Gibberellins
HGT	Horizontal gene transfer
HYDRUS	Hydrus-1D model
IAA	Auxins
LC-HRMS	Liquid chromatography-high resolution mass spectrometry
MDA	Malondialdehyde
MGEs	Mobile genetic elements
MRL	Maximum residue limit
POD	Peroxidase
PSII	Photosystem II
ROS	Reactive oxygen species
SOD	Superoxide dismutase
WHPA	Well head protection area
